# Huntington’s disease from the patient, caregiver and physician’s perspectives: three sides of the same coin?

**DOI:** 10.1007/s00702-012-0787-x

**Published:** 2012-03-08

**Authors:** Krzysztof Banaszkiewicz, Emilia J. Sitek, Monika Rudzińska, Witold Sołtan, Jarosław Sławek, Andrzej Szczudlik

**Affiliations:** 1 Krakowska Akademia Neurologii, Krakow, Poland; 2 Department of Neurology, Medical College, Jagiellonian University, Krakow, Poland; 3 St. Adalbert Hospital, Neurology Gdansk, Gdansk, Poland; 4 Department of Neurological and Psychiatric, Nursing Medical University of Gdansk, Gdansk, Poland

**Keywords:** Disability, Quality of life, Caregiver burden, Huntington’s disease

## Abstract

The aim of this study was to identify determinants of functional disability, patient’s quality of life (QoL) and caregivers’ burden in Huntington’s disease (HD). Eighty HD patients participated in the study. Motor and behavioral disturbances as well as cognitive impairment were assessed using motor, behavioral and cognitive parts of the Unified Huntington Disease Rating Scale (UHDRS); Hamilton Depression Rating Scale was used to assess depression. Disability, health-related QoL and the impact of the disease on the caregivers were assessed using the following methods: UHDRS Functional Assessment Score, SF-36 Scale and Caregiver Burden Inventory. Multiple regression analysis showed that motor disturbances, cognitive impairment, apathy and disease duration were the independent predictors of disability. Depression and cognitive disturbances were the determinants of patient’s QoL, while motor disturbances and depression were the predictors of the caregiver burden. Patient’s disability and QoL as well as caregivers’ burden should be taken into consideration while planning treatment strategy and the results of the present study show that the predictors of those treatment targets are different.

## Introduction

Huntington’s disease (HD) is an inherited progressive neurodegenerative disorder, which affects patients’ cognitive, emotional and motor functions and causes severe disability. Apart from involuntary movements (choreic and dystonic) and bradykinesia, HD is associated with cognitive impairment leading to dementia and a wide range of neuropsychiatric problems, e.g., apathy, depression, anxiety and other behavioral disturbances. Physicians usually focus on motor signs when planning treatment, while the behavioral or cognitive disturbances may influence patient and caregiver’s lives to a greater extent than motor symptoms (Hamilton et al. 2003).

Most of the HD patients are unaware of their involuntary movements, which are apparent to the physician (Snowden et al. 1998; Sitek et al. 2011). The severity of disease may be therefore perceived differently by physicians and patients. The physician assesses the severity of symptoms objectively, while the patient focuses on the subjective perceptions of limitations caused by the disease. Hence, the patient’s point of view may be better expressed by the quality of life (QoL) than disability measures. In addition, the severity of disease from the caregiver’s perspective is affected mainly by the amount of physical and emotional effort invested in the patient’s care (Roscoe et al. 2009).

Previous studies did not provide a comprehensive analysis of predictors of disability (Hamilton et al. 2003; Marder et al. 2000) or QoL (Ready et al. 2008; Ho et al. 2009), but focused rather on a single symptom or a group of symptoms of HD and their significance. The aim of the present study was to identify the predictors of patients’ disability, QoL and caregivers’ burden in Huntington disease.

## Patients

Eighty HD patient-caregiver dyads recruited from the Movement Disorders Clinic of the Department of Neurology, Jagiellonian University Medical College in Krakow (41 subjects) and from the Movement Disorders Outpatient Clinic from St. Adalbert Hospital in Gdansk (39 subjects) volunteered for the study. The recruitment was performed between May 2007 and October 2008. The diagnosis was confirmed by DNA analysis for CAG expansion in htt gene.

Participants were selected from a cohort of patients who participate in the European Huntington Disease Network (EHDN) Registry study (Orth et al. 2011). Registry is a multicenter research observational project, for individuals affected by HD. Inclusion criterion was the adult onset HD in a borderline to advanced stage. All participants provided informed consent for participation in the Registry study. The study was approved by the Central Ethics Commission.

## Methods

In order to assess the implications of the disease from three different points of view, the following aspects were analyzed: functional disability, QoL and caregiver burden. In order to evaluate disability, the Functional Assessment Score (FAS), which is a part of the Unified Huntington Disease Rating Scale (UHDRS), was used (Huntington Study Group 1996). SF-36 Scale was used to assess health-related QoL and Caregiver Burden Inventory (CBI) Scale (Novak and Guest 1989) was used to estimate the impact of the disease on the caregivers.

The severity of HD symptoms was also assessed in each patient. For motor disturbances assessment, UHDRS Motor Examination was used. The scale consists of several items including the assessment of chorea, dystonia, bradykinesia, gait and oculomotor impairment. Cognitive dysfunction was assessed with use of the cognitive part of the UHDRS (consisting of Stroop test, verbal fluency trials and Symbol Digit Modalities Test). Depression was evaluated using Hamilton Depression Rating Scale (HAM-D). The severity and frequency of each behavioral symptom were evaluated using UHDRS Behavioral Assessment. Each behavioral symptom was scored 0 to 4, separately for severity and frequency. Higher scores on the UHDRS motor and behavioral scales and CBI are associated with greater impairment. Higher scores on the UHDRS Cognitive and FAS Scales were related to better cognitive function and less significant disability. Higher scores on SF-36 questionnaire were associated with better QoL.

### Statistical analysis

Simple linear regression analysis was used to assess the contribution of the explanatory variables to FAS, SF-36 and CBI. Separate analyses were performed for each outcome measure. The following variables corresponding to specific HD symptoms were included in the analysis: UHDRS Motor, UHDRS Cognitive, HAM-D, UHDRS Behavioral Assessment subscores separately for apathy, psychotic symptoms (including joint assessment of delusions and hallucinations), irritability, aggression and anxiety. Other factors comprised age, gender, age at disease onset, disease duration, CAG repeat number and duration of education. Bonferroni-adjusted alpha level of 0.003 was used for multiple comparisons.

Significant predictors obtained from the simple regression analyses were included in the forward stepwise regression models that were developed separately for each dependent variable.

The coefficient of determination (*R* ^2^) in the simple analysis as well as beta (β) coefficient in the multiple analyses were interpreted as the measures of contribution of each variable to disability, QoL or caregiver burden. The level of significance for multiple regression analysis was set to 0.05.

## Results

Demographic characteristics as well as motor, cognitive and behavioral UHDRS scores of patients are presented in Table 1. Participants from two study sites did not differ significantly in terms of demographic and illness-related features; therefore, further analysis was performed on the pooled data. Six patients were unable to answer questions of the SF-36 questionnaire.

**Table 1:** Demographic and clinical characteristics of the study group

	Mean (min.–max.)	SD
Age (years)	47.7 (23–76)	13.3
Education (years)	12.4 (7–19)	3.4
Age at onset of motor symptoms (years)	39.0 (21–71)	13.8
Duration of disease (years)	8.3 (0.5–28)	5.7
UHDRS Motor	41.8 (4–93)	22.2
UHDRS Cognitive	106 (0–318)	76.0
HAM-D	9.6 (0–29)	6.7
UHDRS Behavioral (total score)	16.1 (0–55)	11.1
UHDRS Apathy subscore	3.6 (0–8)	2.4
UHDRS Psychotic symptoms subscore	0.14 (0–6)	0.78
UHDRS Anxiety subscore	2.0 (0–8)	2.3
UHDRS Irritability subscore	1.9 (0–4)	1.6
UHDRS Aggression subscore	1.6 (0–7)	2.2

*HAM-D*, Hamilton Depression Rating Scale, *UHDRS* Unified Huntington Disease Rating Scale

### Functional disability

Simple regression analysis showed that UHDRS Motor, UHDRS Cognitive, HAM-D, and UHDRS Behavioral total as well as Apathy subscore and disease duration were correlated with FAS. Irritability, Aggression, Anxiety and Psychotic symptoms subscores were not significantly related to disability (see Tables 2, 3). In the multiple regression analysis, UHDRS Motor, UHDRS Cognitive and UHDRS Apathy subscore and disease duration were the independent predictors of disability (adjusted *R* ^2^ = 0.74 for the model).

**Table 2:** Results of the simple regression analysis

	FAS		SF-36		CBI	
	*R*	*R* ^2^	*R*	*R* ^2^	*R*	*R* ^2^
UHDRS Motor	−0.82*	0.66	−0.38*	0.13	0.58*	0.32
UHDRS Cognitive	0.76*	0.57	0.46*	0.20	−0.35	0.11
HAM-D	−0.43*	0.18	−0.71*	0.49	0.47*	0.21
UHDRS Behavioral (total score)	−0.35*	0.11	−0.58*	0.33	0.34	0.10
UHDRS Apathy subscore	−0.47*	0.21	−0.48*	0.22	0.30	0.07
UHDRS Psychotic symptoms subscore	−0.25	0.05	−0.05	0.01	−0.08	0.01
UHDRS Anxiety subscore	−0.20	0.02	−0.23	0.04	0.19	0.02
UHDRS Irritability subscore	0.02	0.01	−0.24	0.04	0.14	0.01
UHDRS Aggression subscore	−0.19	0.02	−0.28	0.06	0.21	0.03
FAS	–	–	0.46*	0.20	−0.56*	0.30
Disease duration	−0.54*	0.28	−0.18	0.02	0.11	0.01
Number of CAG repeats	−0.24	0.04	0.06	0.01	0.18	0.01
Age	−0.21	0.03	−0.24	0.05	0.01	0.01
Gender	0.13	0.01	−0.02	0.01	0.02	0.02
Age at onset	0.01	0.01	−0.18	0.02	−0.07	0.01
Years of education	0.01	0.01	−0.06	0.01	0.21	0.03

Coefficients of correlation (*R*) and coefficients of determination (*R* ^2^) of each of the predictors were calculated separately for FAS, SF-36 and CBI. Statistically significant values are marked with asterisk

*HAM-D*, Hamilton Depression Rating Scale, *UHDRS* Unified Huntington Disease Rating Scale, *FAS* Functional Assessment Score, *CBI* Caregiver Burden Inventory

**Table 3:** Results of the multiple regression analysis, separately for FAS, SF-36 and CBI

	β	95% CI	*p*
FAS			
UHDRS Motor	−0.55	−0.73 to −0.37	<0.001
UHDRS Cognitive	0.21	0.02 to 0.40	0.031
UHDRS Apathy subscore	−0.15	−0.28 to −0.01	0.031
Disease duration	−0.16	−0.30 to −0.03	0.018
SF-36			
HAM-D	−0.63	−0.80 to −0.45	<0.001
UHDRS Cognitive	0.25	0.08 to 0.43	0.004
CBI			
UHDRS Motor	0.45	0.24 to 0.67	<0.001
HAM-D	0.33	0.12 to 0.55	0.003

*β* beta coefficient, *CI* confidence interval, *HAM-D* Hamilton Depression Rating Scale, *UHDRS* Unified Huntington Disease Rating Scale, *FAS* Functional Assessment Score, *CBI* Caregiver Burden Inventory

### Quality of life

UHDRS Motor, UHDRS Cognitive, HAM-D, FAS, UHDRS Behavioral total score and Apathy subscore were related to QoL in the simple regression analysis (see Table 2). In multiple regression, the independent factors of QoL were the measures of depression and cognitive function (adjusted *R* ^2^ = 0.55 for the model).

### Caregiver burden

In the simple regression analysis, UHDRS Motor, HAM-D and FAS were identified to influence CBI score (see Table 2). UHDRS Motor and HAM-D were the only significant independent factors in the multiple analysis. However, the coefficient of determination for the model was rather low (adjusted *R* ^2^ = 0.39).

## Discussion

The results of the present study show that different symptoms of HD contribute to functional disability, QoL and caregiver burden. Motor symptoms, cognitive impairment, apathy and disease duration seem to influence significantly the functional disability. Depression and cognitive impairment determine the patient’s quality of life, while caregiver burden is mostly influenced by motor symptoms and depression. Due to the co-occurrence of several symptoms in a single patient, the exact contribution of a single symptom on the outcome measures is usually difficult to estimate.

The first study on the discrepancy between patients’ and their family members’ perception of the most disturbing features of HD was published by Stern and Eldridge (1975). According to those authors, physical disturbances were the most disturbing for the affected individuals and those of their family members who are at risk of HD, while dementia and personality change were most disturbing for spouses of HD individuals (Stern and Eldridge 1975). The differences between HD patients, family members and medical professionals’ perception of communication deficits were described by Hartelius et al. (2010). The authors pointed out that the triangular perspective approach provides a complete picture of the difficulties caused by the symptom and helps to provide adequate therapeutic solutions.

The results of the present study are consistent with the previous studies which indicate cognitive impairment, motor disturbances and apathy as significant factors influencing activities of daily living (Hamilton et al. 2003). Disability was also previously associated with the disease duration (Marder et al. 2000).

Huntington’s disease QoL was reported to be related mostly to depression (Ready et al. 2008; Ho et al. 2009), functional capacity (Ready et al. 2008; Ho et al. 2009) and cognitive disturbances (Ready et al. 2008). Some relationship between QoL and apathy as well as irritability was also demonstrated (Ready et al. 2008). The profile of QoL predictors in HD seems to be somewhat different as compared to other neurodegenerative disorders. Depressive symptoms, insomnia and disability were the predictors of low QoL in Parkinson’s disease patients (Karlsen et al. 2000). Interestingly motor signs seem to be less important predictors of QoL in PD (Karlsen et al. 1998, 2000) as well as in our group of HD patients, as motor disturbances were not an independent predictor of QoL.

The caregiver’s burden assessment results in HD have never been reported so far. The influence of disease on the caregivers’ well-being was measured previously using QoL of the caregivers. The analysis of factors which constitute caregivers’ QoL showed that the area of social relationships is significantly more affected in HD as compared to other chronic neurological disorders (McCabe et al. 2009). This may be due to behavioral disturbances which cause social embarrassment of the caregiver and rejection by family members or friends. In another study, caregivers’ QoL was found to be related to functional disability and cognitive disturbances (Ready et al. 2008). Quality of life of the caregivers seems, however, to be a different measure of the disease’s influence on the caregivers than the caregivers’ burden. Physical and emotional strains affect both measures; while in case of patient’s relatives, the former is also influenced by psychological distress caused by fear of being at risk of HD (Hayden et al. 1980). Aubeeluck and Buchanan (2007) pointed out that there may be differences in the emotional burden between those caregivers who are and those who are not spouses of HD individuals. Caregivers who are the spouses of HD patients may experience the feeling of guilt due to being involved in transmission of the disease to their children. They are additionally strained by the responsibility to inform children about their risk of having HD (Hayden et al. 1980). One may speculate that those psychological factors may play a dominant role in the caregivers’ burden since most of the burden’s variance (61%) was not determined by the predictors related to the patients’ symptoms according to our study. Cognitive disturbances in our patients seem to have a minor influence on the caregivers. In contrast, in Parkinson’s (D’Amelio et al. 2009) or Alzheimer’s disease (Razani et al. 2007), cognitive impairment is the key predictor of caregivers’ burden.

This is, to our knowledge, the first study addressing predictors of the patient’s functional disability, QoL and the caregiver burden. However, it also has some limitations. First, the UHDRS Cognitive score is a composite score based on tasks with time constraints, which cannot be treated as a marker of severity of dementia and is to a large extent biased by motor dysfunction even at the preclinical stage (Blekher et al. 2009). Presumably, other aspects of cognitive dysfunction, such as executive dysfunction and memory impairment could be more important from the caregiver’s perspective. Second, due to the large number of the examined factors that also influence each other, the number of patients studied appears to be relatively small. Finally, the relationship of the caregiver to the patient was not analyzed. Supposedly, the perception of the patient by the caregiver may be biased by his/her own genetic status in case of the patient’s offspring.

Since HD patients experience physical as well as psychological constraints, a multidisciplinary and coordinated care should be provided to the patient and his/her caregiver (Veenhuizen and Tibben 2009). Patient’s disability and QoL as well as caregivers’ burden should be taken into consideration while planning treatment strategy. The results of the present study show that the determinants of those treatment targets are different.
